# Adherence of health workers to guidelines for screening and management of cryptococcal meningitis in Uganda

**DOI:** 10.1371/journal.pone.0284165

**Published:** 2023-04-10

**Authors:** Olivie C. Namuju, Proscovia M. Namuwenge, Richard Kwizera, Emmanuel Obuya, Paul Kirumira, Rose Naluyima, Cynthia Ahimbisibwe, JaneFrancis Ndyetukira, Hawa Nakato, Robert Kirungi, Jane Gakuru, Samuel Junju, Edwin Nuwagira, Morris Rutakagirwa, Sara Nsibirwa, Vennie Nabitaka, Elizabeth Nalintya, Edward Mpoza, Conrad K. Muzoora, Abdu K. Musubire, David R. Boulware, David B. Meya

**Affiliations:** 1 Department of Research, Infectious Diseases Institute, College of Health Sciences, Makerere University, Kampala, Uganda; 2 Department of Advanced HIV Disease and Treatment, Ministry of Health, Kampala, Uganda; 3 School of Public Health, College of Health Sciences, Makerere University, Kampala, Uganda; 4 HIV Department, Clinton Health Access Initiative (CHAI), Kampala, Uganda; 5 Department of Medicine and International Health, University of Minnesota, Minneapolis, Minnesota, United States of America; Gulu University, UGANDA

## Abstract

**Introduction:**

Health workers’ failure to adhere to guidelines for screening, diagnosis and management of HIV-associated cryptococcal meningitis (CM) remains a significant public health concern. We aimed to assess adherence to the standards of care and management of HIV patients at risk of CM per the MoH guidelines and assess stock management of CM supplies in the period of January to June 2021 at selected public health facilities (HFs) in Uganda.

**Methods:**

The study employed an observational cross-sectional design to assess the level of adherence of health workers to standards of clinical care and management of HIV positive patients at risk of CM as per the clinical guidelines for Uganda, and stock management of CM supplies in the period of January to June 2021in selected public health facilities. The study team used a survey guide designed by MoH to assess and score the screening, diagnosis and management practices of Health Facilities towards CM. Scoring was categorized as red (< 80%), light green (80%-95%), and dark green (˃95%) in the order from worst to best adherence. The data was transcribed into a spread sheet and analysed using STATA–v15.

**Results:**

The study team visited a total of 15 public health facilities including 5 general hospitals, 9 regional referral hospitals (RRHs) and 1 National Referral hospital (NRH). The mean score for adherence to screening and management of CM for all the combined facilities was 15 (64.7%) classified as red. 10 (66.7%) HFs had not performed a baseline CD4 test for eligible patients within 2 weeks of ART initiation. With regards to treatment, 9 (60%) of the HFs were scored as light green on knowledge of the procedure for reconstituting intravenous Liposomal Amphotericin B. None of the HFs visited had potassium chloride tablets in stock.

**Conclusion:**

Major MoH guidelines are generally not being adhered to by health workers while managing cryptococcal meningitis. It is vital that government and implementing partners regularly support HFs with training, mentorship, and support supervision on CM management to improve adherence to CM screening and treatment guidelines.

## Introduction

The human immunodeficiency virus (HIV) epidemic raised the profile of the genus *Cryptococcus* from being an obscure yeast pathogen to becoming one of the important fungal causes of morbidity and mortality worldwide [1]. The majority of cryptococcal meningitis (CM) cases are found in Sub-Saharan Africa (SSA) mostly caused by *Cryptococcus neoformans*, a pathogenic, encapsulated yeast that mainly infects the central nervous system (CNS). CM is a common opportunistic infection in advanced HIV disease (AHD). It is a deadly invasive fungal infection that continues to affect hundreds of thousands of HIV patients with advanced disease each year and is responsible for an estimated 15% - 20% of all AIDS-related deaths [2]. Current global estimates indicate that approximately 280,000 cases of cryptococcal infection occur in people with AHD worldwide. Overall, 90% of cryptococcal disease in people with HIV occurs in those with CD4 T lymphocyte (CD4) cell counts <100 cells/mm^3^. The incidence of cryptococcal meningitis is observed to decline substantially among people treated on antiretroviral therapy (ART) [3].

The estimated prevalence of cryptococcal antigenemia in ART naïve patients with CD4 below 100 cells/ml is 6% in the world, 3–19% in sub-Saharan Africa (SSA) and 8.2% in Uganda [3]. An estimated 4,000 HIV infected Ugandans develop cryptococcal infection annually in the absence of screening and pre-emptive treatment for asymptomatic cryptococcal infection.

Typically, cryptococcal meningitis is diagnosed by performing a lumbar puncture (LP) with testing of cerebrospinal fluid (CSF) by either India ink microscopy, culture, or cryptococcal antigen (CrAg). Detection of CrAg in whole blood, plasma, serum, and CSF using point of care (POC) tests has revolutionised cryptococcal infection diagnosis with improved diagnostic performance and rapid turnaround time. Routine CrAg screening is recommended in all HIV patients with CD4 less than 100 cells/ml. However, current estimates show that only 20% and 30% of eligible patients are receiving CrAg screening in Uganda and worldwide respectively. Besides, there remains a low index of clinical suspicion for all fungal infections in Uganda [4].

Current treatment guidelines for cryptococcal meningitis recommend first line therapy as seven-days of induction with intravenous (IV) amphotericin B deoxycholate and oral flucytosine among HIV patients [5]. However, in resource limited settings like Uganda where flucytosine is not readily available, the guidelines recommend 14-days of IV amphotericin B deoxycholate and high dose oral fluconazole (800-1200mg a day). CrAg screening and pre-emptive treatment is now a recommendation by the WHO and numerous national HIV guidelines. However, effective implementation of CrAg screening in already overburdened, under resourced HIV clinics in Uganda remains challenging [6].

In 2017, the World Health Organization published guidelines for the management and treatment of advanced human immune deficiency virus (HIV) disease within a public health approach [7]. The guidelines recommend a standardized package of care for people with advanced HIV disease, including cryptococcal meningitis, one of the leading causes of mortality in this population. Implementation of these guidelines by health workers at all HFs in the country should contribute to survival of patients with HIV associated CM. It is therefore crucial that all health facilities (HFs) adhere to standards of care and management of HIV patients with CD4 < 200 cells/μL per the CM guidelines for Uganda in order to reduce the mortality due to HIV associated CM especially when the requisite supplies are available. Uganda National HIV consolidated guidelines[8] recommend CrAg screening in HIV-infected persons with CD4 cell count ≤200 cells/μL, yet its implementation remains a challenge.

In 2021, the Ministry of Health conducted a 1-week training on the guidelines. The components of the training were diagnosis of CM, care and management of CM patients and safe preparation of IV Liposomal Amphotericin.

In the current study we aimed to assess adherence to standards of clinical care and management of HIV positive patients with CD4 < 200 cells/μL per the CM guidelines for Uganda, and evaluate stock management of CM supplies in the period of January to June 2021 at the selected public HFs.

## Materials and methods

### Study design and setting

An observational cross-sectional research design utilizing quantitative methods was used. The rationale of the study design was to conduct an assessment to provide information on adherence of health workers to the guidelines of screening, diagnosis and management of CM patients. In this manuscript, health workers were defined as people who deliver care or provide health care services to the sick persons in a healthcare setting or centres. These included medical officers, clinical officers, laboratory technologists, nurses and counsellors. Data were extracted from records of patients that were diagnosed with CM between the months of January and June 2021 from the 15 selected health facilities around the country. The study was conducted mainly in public hospitals, where the general care is free. These hospitals are expected to handle patients referred from lower-level facilities because they have qualified health workers and specialized equipment needed to provide care. The study team visited selected pilot HFs that offer CM treatment and where capacity was built through training prior to commissioning them to provide the specialized CM treatment.

### Study sample

The assessment was conducted in 15 health facilities around Uganda including 2 general hospitals, 1 national referral, and 12 regional referral hospitals, namely: Tororo General Hospital, Kawolo Referral Hospital, Kiruddu National Referral Hospital, Naguru General Hospital, Hoima Regional Referral Hospital, Kayunga Regional Referral Hospital, Jinja Regional Referral Hospital, Mbale Regional Referral Hospital, Soroti Regional Referral Hospital, Moroto Regional Referral Hospital, Fort Portal Regional Referral Hospital, Mubende Regional Referral Hospital, Arua Regional Referral Hospital, Gulu Regional Referral Hospital, and Lira Regional Referral Hospital.

### Sample size and sampling methodology

The study used a total of 15 health facilities applying a purposive sampling technique because most of these HFs are the regional referrals receiving patients in the 14 main regions of the country. The general hospitals were chosen because they are acting as referral hospitals in those regions and handling the management of patients with cryptococcal meningitis. These health facilities selected had also been earlier trained on the management of the disease.

### Data collection method

Data was collected as part of routine program monitoring during support supervision visits. A checklist (S1 Checklist) was used to obtain information from health workers on CM through interviews and also to extract data from facility records on CM from ART clinic, laboratory, medical wards and pharmacy. The facility records used for data extraction included the ART register, the non-suppressed viral load register, and CrAg registers from the HMIS. The variables of extracted from the registers included; number of CM patients and outcome status of the patients at discharge.

While at the health facility, the study team randomly picked 10–20 patients’ files from the medical wards to check whether; 1. CrAg screening was done at the first point of care (at the Emergency units or OPD) for patients with a CD4<200; 2.a lumbar puncture (LP) was done for patients with a positive serum CrAg, 3. The management of CM was according to set standards, and 4. Investigations for laboratory monitoring were done according to set guidelines. Data on knowledge of management of CM was collected through asking the health workers to review the procedure for reconstitution of infusion Liposomal Amphotericin B (Ambisome), and the side effects of IV Liposomal Amphotericin B and 5FC (flucytosine).

### Data management and analysis

The data were entered and cleaned in an MS excel spread sheet. The main outcome of the study was adherence to guidelines of screening and management of CM in HIV patients with CD4 < 200cells/μl. Screening was defined as any patient seen at the hospital with CD4<200cells/μL or for anyone who exhibited signs and symptoms of CM having a serum CrAg test done. Management was defined as a patient who had a positive serum CrAg treated with fluconazole and having a lumbar puncture done for those who presented with signs and symptoms of CM. other variables included, access to baseline CD4, availability of CM commodities including CrAg, fluconazole, potassium chloride (KCL) (IV), KCL (Tabs), flucytosine, and IV Liposomal Amphotericin B.

Analyses were done using STATA Version 15. Descriptive statistics were used to summarize the main outcomes and the secondary outcomes of the study. Chi-square test was used to assess levels of significance between variable.

### Quality control

After each assessment questionnaires were assessed for completeness. This was done to make sure that the data intended to be collected was complete.

### Ethical statement

The data used for this study was routine program data that was collected by Ministry of Health staff with no identifiers. Therefore, there were no ethical violations.

## Results

### General information about facilities assessed

**Fig 1:**
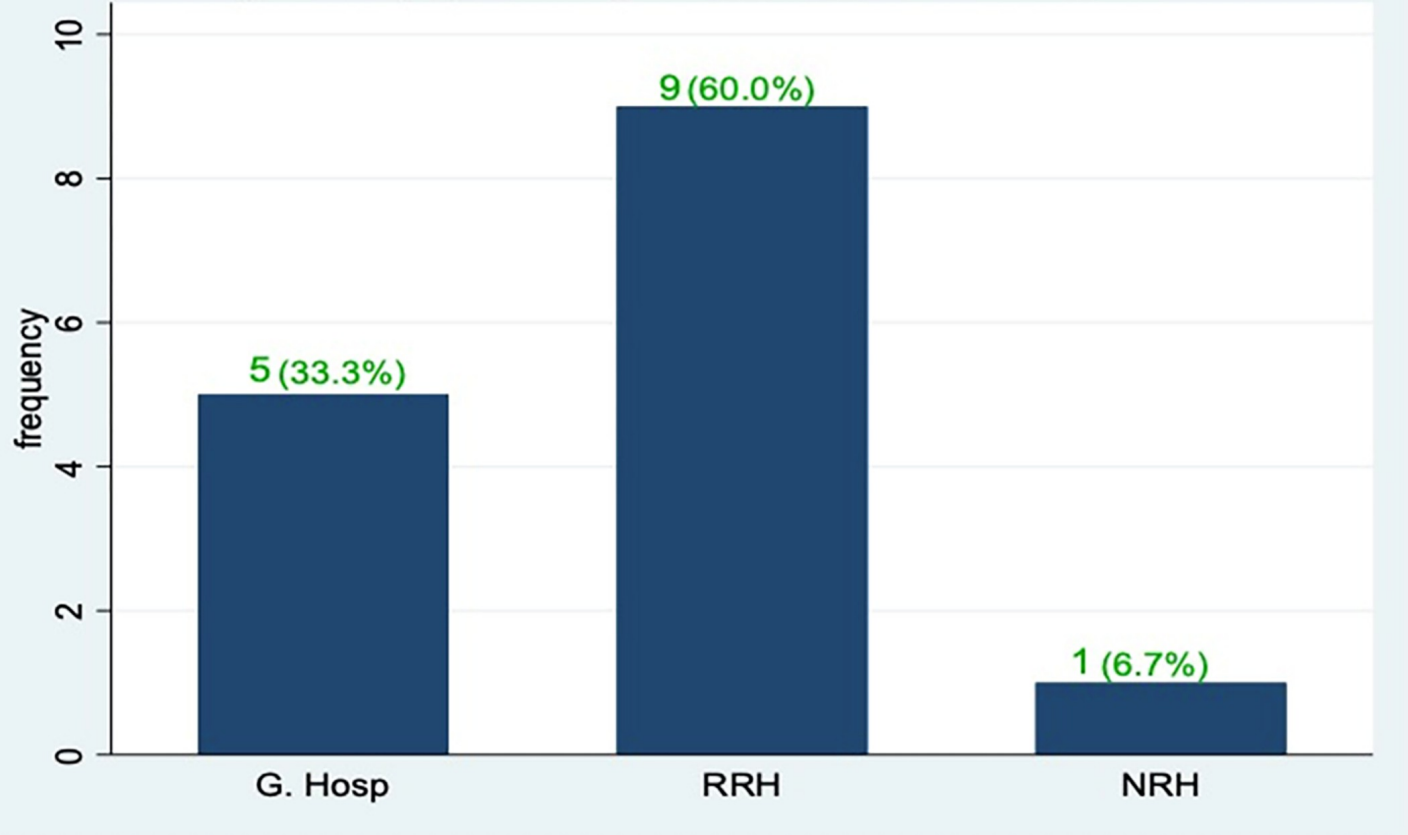
A graph showing the distribution of HFs assessed.

The number of Health facilities (HFs) assessed by this study included 5 (33.3%) general hospitals, 9 (60%) regional referral hospitals and 1 (6.7%) national referral hospital as indicated in (Fig 1). These HFs were chosen purposively on basis of having participated in the baseline training of the management of CM.

### Adherence to screening and management of CM in HIV patients with CD4 count below 200 cells

Results for the assessment of adherence to screening and management of CM are shown in Table 1. The adherence score was calculated as a percentage and graded accordingly as follows: below 80% Red, (80%-95%) light green and above 95% dark green. The mean total score for adherence to screening and management of CM for all the facilities combined was 64.7 with a standard deviation (SD) of 19.7 and a range of (23.5–94.1). This was therefore described as Red. Regarding level of care, the National Referral Hospital visited had the highest mean (SD) score of 94.1, the RRH had a mean (SD) score of 54.9(±21.2) in the range of (23.5–82.4) and the general hospitals visited had a mean (SD) 60.8(±9.3) in the range of (52.9–76.5) (Table 1). This observed difference between different levels of care could be due the availability of research studies ongoing concerning the management of CM as compared to other Health Facilities.

**Table 1:** Summary statistics for total percentage adherence score per facility type.

Facilities	N	Mean	SD	min	Max
**General Hospital**	5	64.7	9.3	52.9	76.5
**Regional referral Hospitals**	9	54.9	21.2	23.5	82.4
**National Referral Hospital**	1	94.1	---	94.1	94.1
**Overall Total**	15	60.8	19.7	23.5	94.5

### Adherence to Uganda National guidelines for cryptococcal antigen screening and CM management

As shown in Table 2, by the time the rapid assessment was done, majority of the HFs, 10 (66.7%) had not done a baseline CD4 200< (within 2 weeks of ART initiation), therefore categorized Red as per the standard of MOH. The national referral hospital visited performed best at dark green, that is above 95% (P = 0.0340) followed by regional hospitals and lastly general hospitals majorly in red. The majority of facilities 14 (93.3%) did not have patients with an unsuppressed viral load (VL) receiving a CD4 count after the date of the viral load test. This finding was uniform across all the levels of the HFs visited with all general hospitals all 8 of the 9 RRHs and the NRH classified as red.

**Table 2:** Standards of screening of HIV positive patients with CD4 below 200 cells.

Variable	Option	Level of Care n (%)			Total n (%)	P-value
		G. Hospital n = 5	RRH n = 9	NRH n = 1		
**A baseline CD4 within 2 weeks of ART initiation**	>95%	1(20.0)	0(0.0)	1(100)	2(13.3)	0.034
	80%-95%	2(40.0)	1(11.1)	0	3(20.0)	
	<80%	2(40.0)	8(88.9)	0	10(66.7)	
**CD4 for patients with Unsuppressed VL**	>95%	0	0	0	0	0.700
	80%-95%	0	1(11.1)	0	1(6.7)	
	<80%	5(100)	8(88.9)	1(100)	14(93.3)	
**Patients with CD4 below 200 cells who had a CrAg test performed**	>95%	0	0	1(100)	1(6.7)	0.005
	80%-95%	1(20.0)	2(22.2)	0	3(20.0)	
	<80%	4(80.0)	7(77.8)	0	11(73.3)	
**Were positive serum CrAg patients given fluconazole/CM treatment**	>95%	2(40.0)	3(33.3)	1(100)	6(40.0)	0.736
	80%-95%	1(20.0)	1(11.1)	0	2(13.3)	
	<80%	2(40.0)	5(55.6)	0	7(46.7)	

From the daily activity register, a record of the number and IDs of patients who had CD4 count less than 200 cells/μL between January 2021 and June 2021. The classification of these numbers was as follows; majority of them were classified as red 11 (73.3%), 3 (20%) classified as light green and only 1 (6.7%) was classified as dark green. Notably, none of the general hospitals and the RRHs visited recorded a dark green.

A look at the CrAg registers or any other improvised tool, assessed if the CrAg positive patients, were initiated on fluconazole/CM treatment and the findings in Table 2 indicated that almost half of the HFs 7 (46.7%) were classified as less than 80% and 6 (40%) classified as 95%. A similar distribution was observed across the levels of the HFs visited even if the probability chi2 was not statistically significant indicating a possible chance distribution (Table 2).

### Adherence to National CM treatment protocols at selected RRH

The study assessed the HF’s record of number of patients treated, discharged with a documented outcome during the study period and the general picture classified to either being red, light green or dark green. The findings in Table 3 showed that there was an equivocal distribution of the grading in the HFs assessed i.e. red and light green had 7 (46.7%) HFs in this grade. None of the general hospitals or the RRHs visited recorded a dark green (p-value = 0.004). The findings on the patients managed showed that 9 (60%) were light green and 6 (40%) were red. After the management, the assessment then requested the health workers to suggest the gaps in the management of CM and notably the unavailability of commodities was the main gap.

**Table 3:** Standards of care and management of HIV patients with CrAg+ and confirmed CM disease.

Variable	Option	Level of Care n (%)			Total N (%)	Probability chi2
		G. Hospital n = 5	RRH n = 9	NRH n = 1		
**Patients diagnosed with CM January—June**	Dark green	0	0	1(100)	1(6.7)	0.004
	Light green	2(40.0)	5(55.6)	0	7(46.7)	
	Red	3(60.0)	4(44.4)	0	7(46.7)	
**Patients managed for CM**	Dark green	0	0	0	0	0.690
	Light green	3(60.0)	5(55.6)	1(100)	9(60.0)	
	Red	2(40.0)	4(44.4)	0	6(40.0)	
**Baseline and monitoring procedures done**	Dark green	1(20.0)	0	0	1(6.7)	0.371
	Light green	1(20.0)	3(33.3)	1(100)	5(33.3)	
	Red	3(60.0)	6(66.7)	0	9(60.0)	
**Reconstitution of IV Liposomal Amphotericin B**	Dark green	2(40.0)	0	1(100)	3(20.0)	0.106
	Light green	2(40.0)	7(77.8)	0	9(60.0)	
	Red	1(20.0)	2(22.2)	0	3(20.0)	
**Assess awareness of side effects of Flucytosine and IV Liposomal Amphotericin B**	Dark green	3(60.0)	2(22.2)	1(100)	6(40.0)	0.191
	Light green	0	5(55.6)	0	5(33.3)	
	Red	2(40.0)	2(22.2)	0	4(26.7)	

The recap on the procedure for reconstituting IV Liposomal Amphotericin B showed that more than half of the HFs, 9 (60%) were classified as light green, 3 (20%) were red and >95% was dark green. The RRHs had the majority of HFs scored as light green 7 (77.8%) and 2 (22.2%) were classified as red (P = 0.106). The assessment on the awareness of common side effects of flucytosine and IV Liposomal Amphotericin B by health workers on medical wards, showed that 6 (40%) were dark green, 5 (33.3%) were light green and 4 (26.7%) were classified as red. Notably, most of the general hospitals visited were dark green (p = 0.191).

### Assessing management of CM stock commodities at the selected RRH

The study reviewed the order forms and delivery notes for the last ordering cycle and commented on order fulfilment for the commodities listed on Table 4. The standard according to this assessment was that commodities ordered are equivalent to commodities delivered. The results categorized as “Yes” or “No” showed that most of the HFs assessed had CrAg tests available 11 (73.3%): general hospitals 4 (80%), RRHs 6 (66.7%) and the NRH visited also had the commodity. Fluconazole was available in 9(60%) of the HFs; 4 (80%) of the general hospitals visited, 5 (55.6%) of the RRHs and not in the NRH visited (P = 0.300).

**Table 4:** Management of CM supplies at the selected HFs.

Variable	Option	Level of Care n (%)			Total n(%)	Probability chi^2^
		G. Hospital n = 5	RRH n = 9	NRH n = 1		
**CrAg**	No	1(20.0)	3(33.0)	0(0.0)	4(26.7)	0.711
	Yes	4(80.0)	6(66.7)	1(100)	11(73.3)	
**Fluconazole**	No	1(20.0)	4(44.4)	1(100)	6(40.0)	0.300
	Yes	4(80.0)	5(55.6)	0	9(60.0)	
**KCL (IV)**	No	5(100)	5(55.6)	0	10(66.7)	0.082
	Yes	0	4(44.4)	1(100)	5(33.3)	
**KCL (Tabs)**	No	4(80.0)	8(88.9)	1(100)	13(86.7)	0.825
	Yes	1(20.0)	1(11.1)	0	2(13.3)	
**Flucytosine**	No	1(20.0)	5(55.6)	0	6(40.0)	0.300
	Yes	4(80.0)	4(44.4)	1(100)	9(60.0)	
**IV Liposomal Amphotericin B**	No	1(20.0)	3(33.3)	0	4(26.7)	0.711
	Yes	4(80.0)	6(66.7)	1(100)	11(73.3)	

Intravenous potassium chloride had been ordered and was not available in all the general and regional hospitals. Potassium chloride tables not available in 4 (80%) of the general hospitals and 8 (88.9%) of the RRHs and none of the hospitals had made an order (P = 0.825).

Flucytosine had been ordered and delivered in 4 (80%) general hospitals, 4 (44.4%) of the RRHs and the NRH. IV Liposomal Amphotericin B had been ordered and delivered in 4 (80%) general 6 (66.7%) of the RRHs and the NRH. All these results were statistically insignificant Table 4.

## Discussion

We have shown from the CrAg register that in the first quarter of 2021 only 19% of those eligible for CrAg screening were actually screened. Of these only 65% of CrAg -positive patients received fluconazole pre-emptive treatment [6]. That shows that there still remains a clear disparity between National guidelines and actual implementation of guidelines at HIV care centres especially at the Regional Referral Hospitals (RRH). Although HFs or Regional Referral Hospitals do a tremendous work of making major contributions to essential clinical care in Uganda, there are questions on whether their care services rhyme rightly with the respective health guidelines of Uganda.

This assessment set out to assess adherence to standards of clinical care and management of HIV positive patients with CD4 below 200 cells per the CM guidelines for Uganda, and stock management of CM commodities in the period of Jan to June 2021 at the selected health facilities. To achieve this, the study assessed adherence to standards of screening of HIV positive patients with CD4 below 200 cells, evaluated adherence to standards of care and management of HIV patients with a positive CrAg test and confirmed CM disease at selected RRH and assessed management of CM stock commodities at the selected HFs.

The NRF outperformed the others dramatically mostly attributed to (1) presence of active on-ground CM related research at the National Referral Facilities; this entails daily reviews, better & robust investigations, timely drug administration among others that translates to high quality clinical care offered by the research team that is experienced in management of AIDS-related CM. (2) Availability of more human resource on ground at NRF, including but not limited to specialized consultant physicians experienced in management of AHD, medical officers, Senior house officers (as the NRF are training hospitals), nurses, Laboratory scientists and technicians etc. Setting up facility specific CQI (continuous Quality improvement) projects related to improving CM screening, diagnosis, management etc. will enable specific facilities troubleshoot problems that hinder performance. A national CQI collaborative can be set up to attain this goal. The observed deficiencies could be due to staff turnover, and knowledge gaps in relation to the comprehensive screening, diagnosis and management of CM. This could also be attributed to knowledge gaps in the ordering of CM related commodities as these are not routinely supplied and have to be separately and specifically requested (CM commodities were previously not on essential medicines list or on the bi-monthly commodities order-list). Our study findings are similar to a study done in South Africa using clinical audits in routine practice, which suggested the presence of critical gaps in clinicians’ adherence to guidelines of CM management [9]. While 66–80% of CrAg tests in stock is not terrible, it likely contributes to the inadequate screening. Certainly, 100% of individuals cannot be screened when only 66–80% of individuals have access to testing.

Results from this assessment have indicated that the health facilities are not currently carrying out baseline CD4 counts within 2 weeks of ART initiation. Only the NRH had a preferable percentage of greater than 95% in the assessment. This finding could be as a result of shortage of trained specialists in the other HFs. This finding was similar to results by Shoham *et al*. [10] who showed the occurrence of deviations from stipulated guidelines. For example, the majority of health facilities performed CD4 counts of patients found to have an unsuppressed viral load as stated in the existing guidelines.

The majority of health facilities scored red in the area of having updated records. Health facilities did not have updated records of the numbers and IDs of patients with CD4 cells less than 200 cells. This result was consistent with findings of Rajasingham [3]. Results from assessment of the CrAg register showed that almost half of the HFs scored dark green, which should be maintained and would probably lead to improved management of patients if adopted across all the HFs. Okwir *et al*. similarly found that proper record keeping improves management of CM patients. [11]. There is need to educate healthcare providers on the need to consistently and accurately document records.

With regards to the management of CM, that is the number of patients treated and an outcome documented in the study period, the results showed that all HFs scored within light green and red and none of the health facilities attained the highest score of dark green. This finding was different from that of Ford *et al*. [12] who showed that it is imperative to implement the package of CM management as recommended by WHO.

IV Liposomal Amphotericin B, one of the drugs used in the treatment of CM, requires reconstitution before it can be administered to patients. The majority of HFs were able to perform the procedure adequately well, scoring more than 60%, however, this could be improved. This finding was similar to that of Shoham *et al*. [10] who made a recommendation of further follow-up on reconstitution.

The study also assessed for availability and accessibility of drugs, testing kits, and reagents that would be required in the diagnosis and management of CM. Most of the HFs had IV Liposomal Amphotericin B, testing kits, and reagents, however, fluconazole, flucytosine and KCL tablets were not readily available. The reason for this observation is that these drugs are quite expensive and cannot be procured privately by the HFs while the National Medical Stores, which is responsible for suppling medications to the public HFs dose not stock some of these drugs for example KCL tablets.

This study had major strengths—we used guidelines recommended for use in the management of cryptococcal disease by MOH for computing the adherence of CM management. The study team managed to reach out to the major referral hospitals in the country that are expected to be screening and treating patients with cryptococcal disease. One weakness was that a review of the hospital records was used and this may not have been an accurate record so there was no way of measuring proper follow up of the patients observed. There is a probability of information bias due to the tools used for data collection either by the study or hospital staff who document data into the log records or registers.

### Study limitations

Not all the regional referral hospitals were assessed, so the data may not be representative of the whole country. Since the cross-sectional designs are a snapshot nature of studies, they do not give a good basis for establishing causality of the problem because data collection is done in an easy and quick way. Due to the short duration of the study and its timing, data may not be representative too.

## Conclusion and recommendations

This study showed that the adherence to CM management was below the recommended level per Ministry of Health Uganda. Health workers in public HFs across the country are not adherent to clinical guidelines of screening, diagnosis and management of CM as recommended by MOH. Some of the relevant drugs required for CM management were generally not available in the public health facilities.

HFs and RRHs need more regular and enhanced support with training and mentorship programs on screening, diagnosis and CM management. Managers of HFs should be encouraged to implement and supervise the use of clinical guidelines in the management of diseases as recommended by MOH. Ongoing dialogue between the healthcare providers, MOH and NMS is warranted to ensure that all the relevant drugs and test kits required for the management of CM patients, are procured and delivered in adequate amounts to all the HFs across the country. Further studies to assess adherence to clinical guidelines for Advanced HIV Disease management as a pathway to improve survival are needed.

## Supporting information

S1 Checklist Interview guide for the study. (DOCX)

S1 Dataset Raw data set for the study. (XLSX)
