# Supplementary material for: Adherence of health workers to guidelines for screening and management of cryptococcal meningitis in Uganda
Source: PLoS One. 2023 Apr 10;18(4):e0284165. doi: 10.1371/journal.pone.0284165 (PMC10085016; doi:10.1371/journal.pone.0284165)
Supplement: S1 Checklist — (DOCX) [file pone.0284165.s001.docx]

**MINISTRY OF HEALTH**

**AIDS CONTROL PROGRAM**

**SUPPORT SUPERVISION TOOL FOR CRYPTOCOCCOCAL MENINGITIS TREATMENT SITES**

**Background**

The government of Uganda, through Clinton Health Access Initiative, Inc. (CHAI), received funding from Unitaid to increase the country’s capacity to screen and manage advanced HIV disease. The Unitaid Advanced HIV Disease (AHD) initiative aims to reduce morbidity and mortality by accelerating access to optimal products for the prevention and management of key opportunistic infections.

The Ministry of Health, in collaboration with CHAI, is working to improve the capacity of selected hospitals to diagnose and treat Cryptocococcal Meningitis (CM). To this end, training were conducted at targeted hospitals in November and December last year. The aim of the training was to equip trainees (doctors and nurses) with the knowledge and skills to:

a. Appropriately screen patients for CM and other neurological manifestations of HIV

b. Use patient risk factors, and results of a lumbar puncture to distinguish between the common pathogens associated with meningitis

c. Design a tailored treatment regimen and monitoring plan for a patient with CM

d. Design a tailored treatment regimen and monitoring plan for a patient with other forms of neurological manifestations of HIV

e. Appropriately provide secondary prophylaxis against infections for eligible patients.

This post training support supervision is planned at the site to review progress toward the above objectives and to support health workers to address any gaps identified.

**Specific Objectives of the support supervision**:

1. To assess adherence to standards in AHD screening among eligible patient categories (i.e. New patients, non-suppressed, those returning to care and children)
2. To assess adherence to standards for management of Cryptococcocal meningitis including treatment monitoring
3. To assess stock management for Ambisome and Flucytosine
4. For Naguru, Kirruddu, Mubende, Mbarara, Masaka hospitals work with the team to replace faulty filters co-packaged with the batch **D2000004D of** Ambisome. Refer to SOP attached for this activity and al members involved should sign to confirm adherence to the SOP.

| Name of Hospital | Jinja Regional Referral Hospital |
| --- | --- |
| Date of Supervision | 24^th^ /09/2021 |
| Supervisors | |
|  |  |
|  | Dr. Gakuru Jane |
|  | Ndyetukira Jane Frances |
|  | Ian Nyamitoro |
|  |  |
| Brief summary:   1. Number of attendees 2. Key gaps identified and remedies | Number of attendees - 20  Key Gaps   1. No reagents to do CD4 count. 2. No TB LAMs, CrAg strips. 3. No CrAg register |

| **STANDARD 1: All new PLHIV newly initiating on ART or have unsuppressed VL should get a CD4+ test. Patients with CD4+ less than 200 should be screened for TB and Cryptococcal Meningitis.**  ***Note: This should be assessed at the ART/TB clinics*** | | | |
| --- | --- | --- | --- |
|  |  | Response | Scoring |
| Q1 | Instructions: From the **ART register**, randomly select 20 patients who initiated ART between Jan and June 2021. Record the number of patients who received a baseline CD4+ (within 2 weeks of ART initiation). Calculate percentage. | Red- 35% | <<80%=Red  80-95%=Light green  >95%=Dark green |
| Q2 | Instructions: From **the unsuppressed register**, randomly select 20 patients who had an unsuppressed VL between Jan 2021 and June 2021: Record the number of patients who received a CD4+ after the date of the unsuppressed VL. Calculate percentage. | Red- 65% | <<80%=Red  80-95%=Light green  >95%=Dark green |
| Q3 | Instructions: From the daily activity register, record the number and IDs of patients who had CD4+ less than 200cells/dl between Jan 2021 and June 2021. Out of these record the number and percentage of patients who received;   1. Both urine TB LAM test and a CRAG test 2. Only TBLAM test 3. Only CRAG test | Red | If option 1 is 100% then Dark green otherwise = Red |
| Q4 | From Daily activity register, determine number of CrAg positive patients identified from between Jan 2021 and June 2021. Ask for a CRAG register or any other improvised tool, to assess if the CrAg positive patients, were initiated on fluconazole/CM treatment? Calculate percentage. | Dark Green | <80%=Red  80-95%=Light green  >95%=Dark green |
| Q5 | From Daily activity register, determine number of TBLAM positive patients identified between Jan 2021 and June 2021. Check in the unit TB register, to assess if they were initiated on TB treatment? Calculate percentage | Dark Green | 80%=Red  80-95%=Light green  >95%=Dark green |
| Comments:   1. No; CD4 re-agents, TB LAM, CrAg strips as well. 2. They don’t have CrAg register. | | | |

| **Standard 2: All patients with CM meningitis should be treated with optimal treatment regimens** | | | |
| --- | --- | --- | --- |
| Q6 | From the male and female ward, record the number of patients treated for CM meningitis between December 2020 and August 2021.   \| Month \| Dec 2020 \| Jan 2021 \| Feb 2021 \| Mar 2021 \| April 2021 \| May 2021 \| June 202 \| July 2021 \| August 2021 \| \| --- \| --- \| --- \| --- \| --- \| --- \| --- \| --- \| --- \| --- \| \| Number treated \| 0 \| 3 \| 0 \| 4 \| 3 \| 3 \| 1 \| 1 \| 0 \| \| Number discharged \| 0 \| 3 \| 0 \| 4 \| 1 \| 3 \| 1 \| 0 \| 0 \| \| Number of mortalities \| 0 \| 0 \| 10 \| 0 \| 1 \| 0 \| 0 \| 1 \| 0 \| | | |
| Q7 | From the patients above, select at least 10 files for further assessment as follows:  -Four files provided   \| Induction phase drugs \| Number of patients \| Comments \| \| --- \| --- \| --- \| \| Amphotericin B liposomal (3mg/kg/day)/ deoxycholate (1mg/kg/day) + Flucytosine (100mg/kg/day in 4 divided doses) for 1 week, followed by 1 week of fluconazole (1200 mg/day for adults, 12 mg/kg/day for children and adolescents). \| 4 \| Drugs are available \| \| Fluconazole (1200 mg daily for adults, 12 mg/kg/day for children and adolescents) + Flucytosine (100 mg/kg/day, divided into four doses per day. \| 0 \|  \| \| Amphotericin B deoxycholate (1mg/kg/day) + high-dose Fluconazole 1200mg/day. \| 0 \|  \|   Note: Only 4 files provided  For each patient, indicate if they received baseline and monitoring procedures below:   \| Parameter \| 1 \| 2 \| 3 \| 4 \| 5 \| 6 \| 7 \| 8 \| 9 \| 10 \| \| --- \| --- \| --- \| --- \| --- \| --- \| --- \| --- \| --- \| --- \| --- \| \| Number of LPs during treatment \| 1 \| 1 \| 1 \| 1 \|  \|  \|  \|  \|  \|  \| \| Baseline CBC \| 1 \| 1 \| 1 \| 1 \|  \|  \|  \|  \|  \|  \| \| Baseline RFTs \| 1 \| 1 \| 1 \| 1 \|  \|  \|  \|  \|  \|  \| \| Repeat CBC \| 1 \| 0 \| 0 \| 0 \|  \|  \|  \|  \|  \|  \| \| Repeat RFTs \| 1 \| 0 \| 0 \| 0 \|  \|  \|  \|  \|  \|  \| \|  \|  \|  \|  \|  \|  \|  \|  \|  \|  \|  \| | | |
| Q8 | In case of gaps: Ask health workers to explain the reasons for the gaps:   1. LPs not accepted/ runaway 2. CrAg strips and Urine LAM not available 3. CSF chemistries not being done 4. Lab results take long to return 5. No LP needles on the lab. | | |
|  |  | Response | Scoring |
| Q9 | **Instructions:** Request at least 2 health workers (preferably nurse) to recap the procedure for reconstituting Ambisome. | Light green | If aware not aware =Red  If aware of but has some gaps =Light Green  If aware of entire procedure >=Dark Green |
| Q10 | **Instructions**; Assess awareness of common side effects of Flucytosine and Ambisome by health workers on medical wards:  Ambisome:   1. Infusion rigors 2. Hypokaelemia 3. Renal insufficiency   Fluocytosine:   1. Neutropenia | Dark green | If aware of none=Red  If aware of 1=Light Green  If aware of all >=Dark Green |
| Comments:   1. More training on Amphotericin B administration required. | | | |
| 1. Train on any areas where gaps are noted. Outline the areas trained on below:   …Revisited CM management and administration of Amphotericin B………………………………………………………………………………………………………  ………………………………………………………………………………………………………………  …………………………………………………………………………………………………………… | | | |
| 1. Demonstrate procedure for estimating CSF opening pressure without a manometer:   Done | | | |
| 1. Review CM treatment form and patient card   Done | | | |

**AHD COMMODITY MANAGEMENT**

| **AHD and CM Commodity Security Order fulfilment**  Review the order forms and delivery notes for the last ordering cycle and comment on order fulfillment for the commodities below: | | | | |
| --- | --- | --- | --- | --- |
| **Standard;** commodity ordered equals commodities delivered | | | | |
| **Commodity** | **Quantities ordered** | **Quantity received** | **Stock on hand** | **Expiry date** |
| CD4+ Reagents | **1000** | **0** | **0** |  |
| LAM kits | **1000** | **0** | **0** |  |
| CrAg | **1000** | **0** | **0** |  |
| Fluconazole |  | **0** | **0** |  |
| KCl (IV) |  |  | **0** |  |
| KCl (tabs) |  |  | **Not stocked** |  |
| Comment on stock on hand and average month consumption for catalytic procurement | | | | |
| Commodity | **Current stock on hand (Full tins for Flucytosine, vials for Ambisome)** | **Average Monthly Consumptiom** | **Stock on Hand** | **Expiry Date** |
| Flucytocine | **0** | **300** | **0** |  |
| Ambisome | **0** | **41** | **0** | **2/23** |
| Comment on record keeping in general   1. Last received lab supplies in December 2020. 2. 25 vails of Ambisome and 80 tabs of flucytosine available at the dispensary. | | | | |

**REPLACEMENENT OF DEFECTIVE FILTERS CO-PACKAGED WITH AMBISOME**

For Mubende, Mbarara, Masaka hospitals work with the team to replace faulty filters co-packaged with the batch D2000004D of Ambisome. Refer to SOP attached for this activity and all members involved should sign to confirm adherence to the SOP provided.
